# Adherence to the Mediterranean Diet in Association with Self-Perception of Diet Sustainability, Anthropometric and Sociodemographic Factors: A Cross-Sectional Study in Italian Adults

**DOI:** 10.3390/nu13093282

**Published:** 2021-09-20

**Authors:** Beatrice Biasini, Alice Rosi, Davide Menozzi, Francesca Scazzina

**Affiliations:** 1 Department of Food and Drug, University of Parma, Via Volturno 39, 43125 Parma, Italy; beatrice.biasini@unipr.it (B.B.); alice.rosi@unipr.it (A.R.); 2 Department of Food and Drug, University of Parma, Via Kennedy 6, 43125 Parma, Italy; davide.menozzi@unipr.it

**Keywords:** Mediterranean Diet, sustainable diet, food frequency questionnaire, diet self-perception, socioeconomic profile, health status, adult population

## Abstract

The adoption of sustainable dietary models, such as the Mediterranean Diet (MD), can be a valuable strategy to preserve ecosystems and human health. This study aims to investigate in an Italian adult representative sample the adherence to the MD and to what extent it is associated with the self-perceived adoption of a sustainable diet, the consideration of the MD as a sustainable dietary model, and anthropometric and sociodemographic factors. By applying an online survey (*n* = 838, 18–65 years, 52% female), an intermediate level of MD adherence (median: 4.0, IR: 3.0–4.0) in a 0–9 range was observed. Only 50% of the total sample confirmed the MD as a sustainable dietary model, and 84% declared no or low perception of adopting a sustainable diet. Being female, having a higher income and education level, considering the MD as a sustainable dietary model, as well as the perception of having a sustainable diet were the most relevant factors influencing the probability of having a high score (≥6) of adherence to the MD. This study suggests a gradual shift away from the MD in Italy and supports the need to address efforts for developing intervention strategies tailored to adults for improving diet quality. Furthermore, a public campaign should stress the link between a diet and its environmental impact to foster nutritionally adequate and eco-friendly dietary behaviors.

## 1. Introduction

Dietary patterns and the frequency of food consumption are associated with a person’s health status, but they also substantially modulate resource exploitation. It has been largely proven that several health advantages can be linked to the adoption of a dietary pattern inspired by the Mediterranean Diet (MD) [1,2]. A Mediterranean-type diet is able to prevent the development of cardiovascular disease—not only in populations living in the Mediterranean area [1,3,4]—reducing the risk of diabetes [5] and metabolic syndrome [6,7]. In addition, an inverse association between adherence to the MD and the risk of several cancer types and cancer mortality [8], depression, and cognitive impairment [9] has been demonstrated, with beneficial effects on sleep quality in the adult population [10].

The MD is a nutritionally adequate plant-centered diet whose pillars are similar to those qualifying the global “healthy diet from sustainable food systems” described by the EAT Lancet Commission [11]. It is grounded in the “One Health” concept, strengthening the idea that human and ecosystem health are not independent [12,13]. Both dietary approaches largely include whole grains, fruit and vegetables, nuts, and unsaturated fatty acid sources (e.g., olive oil), and both limit the consumption of red meat, processed meat, starchy vegetables, added sugar, and refined grains. However, a stricter limitation is addressed to fish and white meat intake in the reference diet described by the EAT Lancet Commission [11,14]. If food security, affordability, accessibility, and cultural acceptability are ensured, the MD emerges as an example of a sustainable dietary pattern able to address health and ecological concerns [15,16], showing a lower environmental impact, richness in biodiversity, important food-related sociocultural values, and positive economic return for local communities [17]. As found in previous studies, the dietary shift toward a Mediterranean-type diet can lead to positive outcomes both for health and climate change [18,19].

It is worth noting that the MD, acknowledged by UNESCO as an intangible cultural heritage of humanity, should not be considered just as a mere reference from which a particular set of food needs to be consumed in specified quantities and proportions, but also as a cultural model involving not only consumption, but food production, processing, distribution, and cooking, including rituals and traditions [14,20]. On the basis of the health-promoting outcomes linked to the adoption of a Mediterranean dietary pattern, scientific efforts have been increasingly addressed to develop proper methods to estimate the adherence to the MD by using indexes or scores and to test for their disease risk predictive ability [21]. As a result, such indexes or scores can be calculated from the intake of certain food components and lifestyle factors [22].

In the last few decades, Mediterranean populations, including Italian adolescents [23] and adults [24], are stepping away from their traditional dietary patterns, such as the MD. Important determinants are globalization, which overcomes geographical barriers between food productions and consumption worldwide, population growth, urbanization, and lifestyle changes, together with economic and sociocultural factors [15,25]. Food system and food consumption pattern transformations are responsible for a loss of biodiversity and soil degradation and represent significant challenges for the state of food security and nutrition [26].

Based on these considerations, the aim of the present study was to investigate the adherence to the MD and to what extent this variable is associated with the self-perception of the MD’s sustainability, as well as the adoption of a sustainable diet in a representative sample of Italian adults. The secondary aim was to explore how demographic, socioeconomic, and behavioral variables may influence adherence to the MD.

## 2. Materials and Methods

### 2.1. Sample

After receiving approval from the local institutional ethical committee (Comitato Etico Area Vasta Emilia Nord, 1139/2018/OSS/UNIPR), an online survey instrument was distributed to a representative sample of adults residing in Italy (18–65 years) through a software platform from a marketing agency (Qualtrics International Inc., Seattle, Washington and Provo, Utah, United States of America) in July 2019. The agency invited subjects to participate by sending communication via e-email to pre-enrolled members. The survey, which is part of a PhD thesis [27], was completed on one occasion by the respondents, who provided their informed consent to the study. Only the subjects living in Italy and not affected by cardiometabolic conditions, such as diabetes and cardiovascular diseases, as well as eating disorders were eligible to participate in the present cross-sectional study. The data were collected from subjects representative of the adult population residing in Italy based on three selected criteria: gender distribution, age range, and geographical areas of residence (nomenclature of territorial units for statistic (NUTS 1)). When a sufficient number of individuals completed the survey, subject recruitment and online survey self-administration was stopped. The enrolled participants received compensation after the survey’s completion. To be representative of the entire Italian adult population (*n* = 37,248,990, as indicated by the dataset provided by the National Institute of Statistics (ISTAT), referred to on 1 January 2019), the minimum sample size was set at 666 participants, taking into account a confidence level of 99% and confidence interval of 5%. As some participants could drop out during the study or could be excluded from the analyses because of missing answers, more than 800 respondents were invited to participate to the study. The calculation of the sample size was performed using the sample size calculator suggested by the National Institute of Health (https://www.epicentro.iss.it/strumenti/SampleSize, accessed on 30 June 2021).

### 2.2. Measures

Anthropometric and sociodemographic information was self-reported by the subjects. Height and weight were assessed as continuous variables, while others were assessed as categorical variables, including sex and nationality (2 categories each), educational attainment and income (3 categories each), size of residence, number of members and children within the household (4 categories each), age, occupation, and geographical area of residence (5 categories each). Using weight and height data, the subjects’ BMIs were computed, and weight status was defined by applying the WHO’s standard cut-offs [28]. The subjects were asked to express the degree of responsibility for food purchasing and meal preparation (e.g., being the main person responsible, co-responsible, or little or not at all responsible), the habitual frequency of eating out (e.g., never or seldom, <1 time/week, 1 time/week, 2–4 times/week, or ≥5 times/week), the presence of a certain physiological status (e.g., pregnancy or breastfeeding), particular health risk factors (e.g., hypertension or dyslipidemia), and food allergies or intolerances. In addition, the respondents were asked to indicate any participation in environmental associations (EAs) or solidarity purchasing groups (SPGs), which are local networks of people who organize collective purchase decisions regarding food and other goods, selecting suppliers based on critical consumption and solidarity criteria. Specifically, SPGs are intended to promote environmental sustainability (e.g., selecting seasonal, organic, or local products) and social sustainability with respect to the producers (by creating social bonds with them) and SPG members themselves (e.g., by providing mutual assistance) [29,30].

Adherence to the MD was assessed by using a 15-item food frequency questionnaire, already validated to determine the adherence to the Mediterranean dietary pattern of Italian adults on a score from 0 (minimal adherence) to 9 (maximal adherence) [31]. The scoring scheme of the components was binary, with a score of 0 or 1 being associated to the intake of each included food group or item, expressed as the number of portions per day or week. In detail, 1 point was assigned as follows: vegetables (≥2/day), fresh fruit (≥2/day), dried fruit (≥2/week), wholegrain cereals (≥1/day), pulses (≥2/week), fish (≥2/week), olive oil (≥3/day), red and processed meat (<1–3/week), and wine (1–2 glasses per day for men and less than 1 glass per day for women). The final score was computed by summing each individual score. In addition, according to the obtained level of adherence (tertiles) to the MD, the respondents were divided into low (fist tertile, MD score 0–2), medium (second tertile, MD score 3–5) and high (third tertile, MD score 6–9) adherence groups. Moreover, the level of adherence to the MD was evaluated according to the subjects’ compliance with the Italian national recommended intake for the food groups or items considered for a standard dietary energy intake of 2000 kcal/day [32]: fruits (3/day), vegetables (≥2/day), nuts (2/week), legumes (3/week), red meat (≤1/week), fish (2–3/week), wine (never or hardly ever), white meat (2/week), sweets (<1/week), butter, margarine, or cooking cream (≤3/day), olive oil (3/day), milk or yogurt (3/day), and carbonated or sugar-sweetened beverages (<1/week).

After providing the respondents with the definition of sustainable diets expressed by the Food and Agricultural Organization (FAO) [33], including a more explicit and concrete description to provide respondents with a unique interpretation, the online survey also included a question addressed to understanding if the respondents perceived the MD as a sustainable diet (i.e., “Do you think that the MD can be considered a sustainable dietary model?”). This item was adapted from those developed by Riddell et al. [34] for measuring the self-perception of diet and healthy eating. In addition, the self-perceived adoption of a sustainable diet during the last 3 months was assessed (i.e., “I can say that I have adopted a sustainable diet within the last 3 months”) as adapted from Fishbein and Ajzen [35]. Both the answers were measured on a unipolar 7-point scale anchored by totally disagree or totally agree answers. However, for reasons pertaining to result interpretation, the answers were collapsed into 3 categories: “no”, including those who *disagreed* or *totally disagreed*; “not much/maybe”, corresponding to those who *somewhat disagreed*, were *neutral*, or *somewhat agreed*; and “yes”, referring to those who *agreed* or *strongly agreed*.

### 2.3. Data Analysis

Descriptive and inferential statistics were collected. Normality of the data distribution was rejected through the Kolmogorov–Smirnov test. The results were expressed as a frequency (%) or as median and interquartile ranges. The Chi-square test (*χ*^2^) was used to explore potential associations between gender and (1) demographic and socioeconomic characteristics, (2) adherence to the MD, (3) the perception of the MD as a sustainable dietary model, and (4) self-perceived adoption of a sustainable diet. The same test was applied also to investigate potential associations between adherence to the MD (with the subjects divided in MD score-based tertiles) and frequency of food consumption recommended by the Italian dietary guidelines [32]. The non-parametric Mann–Whitney test was applied to explore differences between genders (males and females). Furthermore, to evaluate which characteristics were able to predict scores of high adherence to the MD, univariate and multivariate logistic regression analyses were carried out. A *p*-value less than 0.05 was considered statistically significant. All statistical analyses were performed with IBM SPSS Statistics for Windows, version 25.0 (Armonk, NY: IBM Corp).

## 3. Results

### 3.1. Participants’ Characteristics and Adherence to the MD

Overall, 860 subjects answered the online survey. After removing the low-quality records (*n* = 22), the final sample was composed of 838 respondents, representative of the adult residents in Italy. The participants’ characteristics are provided in Table 1. Approximately half of the respondents were females (52%), and most of the respondents were from 35 to 65 years old (71%). The vast majority of the sample attained at least secondary education (79%). Different employment conditions were observed by comparing males and females, with more than two-thirds of males working as full-time employees and more than one-fourth of females being unemployed. Most of the respondents were apparently healthy, declaring a normal body weight; however, a significant association was found when the sample split by gender was grouped by BMI categories, with a higher proportion of males being overweight or obese compared with females (*p* < 0.001). Moreover, a higher percentage of females compared with males had the main responsibility of food purchasing and meal preparation.

After dividing the respondents according to their level of adherence to the MD, an association between gender and the level of adherence to the MD was spotted (*p* < 0.001). A medium compliance to the MD was reported, with females presenting significantly higher scores compared with males (*p* < 0.001).

Although only 1% of the subjects clearly disagreed in considering the MD as sustainable, half of the sample (49%) expressed uncertainty with this statement. On the other hand, a low number of respondents (16%) (strongly) agreed to having adopted a sustainable diet in the last 3 months.

### 3.2. Compliance with Food Recommendations

Table 2 shows the number and proportions of respondents being compliant with the consumption frequency or frequencies used to compute the MD score or those recommended by Italian guidelines. Significant associations were found between the MD adherence categories (low, medium, and high) and all the food items or groups used to compute the final MD. In addition, the level of adherence to the MD was found to be in association with being compliant with the Italian national recommended intake for fruit, vegetables, milk and yogurt, red meat, carbonated or sweet beverages, fish and seafood, nuts, and pulses.

Irrespective of the level of adherence to the MD, the food frequency questionnaire provided a qualitative descriptive picture of respondents’ dietary habits (Supplementary Table S1). More than half of the subjects indicated they ate one or less than one portion per day of fruits (59%) or vegetables (69%). Similarly, 61% and 55% of respondents stated they consumed less than one portion per day of wholegrain pasta or rice as well as bread or its substitutes, respectively, with approximately 20% of the whole sample never or hardly ever eating wholegrain products. More than 50% of people stated that they instead consumed from 1 to 3 portions a week of red or white meat, while 9% consumed 4 or more portions of red meat per week. Furthermore, 72% of the respondents consumed no more than 1 portion per week of legumes.

### 3.3. Associations between Adherence to the MD and Anthropometric, Sociodemographic, and Sustainability Perception of Diet Variables

Among the factors influencing the probability of having a high score of adherence to the MD, being female, having a higher income and educational level, considering the MD a sustainable dietary model, as well as perceiving having a sustainable diet were the most relevant, as they were found to be statistically significant in the univariate and multivariate regression analysis (Table 3). Other conditions such as being overweight or obese and having no or little responsibility in food purchases or meal preparation significantly decreased the probability of having a high adherence level to the MD only when tested singularly in the univariate analysis, suggesting its being less incisive in affecting this outcome. An opposite effect instead was found for pregnancy, breastfeeding, experiencing menopause, and living in a big city (n. of inhabitants > 500,000), which positively influenced the adherence score, according to the same analysis.

## 4. Discussion

This cross-sectional study gives information on the adherence to the MD in a representative sample of adults residing in Italy. Furthermore, this investigation sheds light on the subjects’ evaluation of the Mediterranean dietary model as a sustainable diet based on the FAO’s statement [33] and on whether the subjects perceived their dietary habits of the last 3 months to be sustainable. These two variables have not been explored in relation to the Mediterranean dietary model before, opening the way to further investigations on the relationship between the adoption of a Mediterranean dietary pattern and its subjective interpretation. In fact, to the best of our knowledge, this is the first time in which an association between the adherence to the MD and its evaluation as a sustainable dietary model, as well as between the adherence to the MD and the self-perception of dietary habits as sustainable, has been assessed. Indeed, exploring both consumers’ diets and their perceptions about food consumption is a valuable approach to define effective strategies to opportunely shift dietary behavior in a desired direction. Specifically, the mechanism able to explain the association between self-perceptions of the MD’s sustainability and the adoption of a sustainable diet with the adherence to the MD can reasonably rely on the consumers’ awareness of their own dietary behaviors, their knowledge of food and environmental sustainability issues—concepts largely misunderstood by the general population [36]—and on the MD’s relevance in terms of sustainability. Nevertheless, further research on the potential mechanisms involved in this association are needed. In our study, the MD has been scarcely recognized as a sustainable diet. Thus, given the growing focus and interest on the need to adopt sustainable behaviors and a positive attitude toward food sustainability [36,37], not considering the MD as a sustainable diet could limit the adherence to the MD itself.

In general, a medium adherence to the MD was reported, in line with another recent investigation on Italian adult populations [38]. Similar to what was found by Dinu et al. [38], better adherence to the MD was found in females compared with males. In addition, our results confirm the influence of the educational level, as less educated people showed lower adherence. However, contrary to what was shown by Dinu et al. [38] and by Caparello et al. [39], no associations were observed for age, nor between the compliance to the Mediterranean dietary model and the geographical area of residence. Such discrepancies with our results may reflect the lack of subjects’ representativeness based on the age and geographical distribution of the participants in both of these studies. Our findings reflect the effects of globalization, which has shaped the dietary habits of people living in the Mediterranean Basin toward food consumption that traditionally has characterized non-Mediterranean countries [40]. However, conflicting data on trends of adherence to the MD have been reported. Indeed, while a cross-sectional investigation in South Italy, one of the MD cradles, found a significant decrease in adherence to the MD from the 1980s to the 2000s, mainly in younger groups [41], a study carried out with an adult population living in the north of Italy did not report a significant change in MD adherence from 1991 to 2006 [42].

Socioeconomic status emerged as a factor impacting participants’ dietary habits. Indeed, people declaring higher incomes and education showed better compliance to the Mediterranean dietary pattern, contrary to those in the opposite conditions. Moreover, having a higher income clearly increased the probability for respondents obtaining a higher score of adherence to the MD. This data are confirmed by a recent previous work, which found that a less advantageous socioeconomic status represents an obstacle to following the MD [43]. Although it has been argued that, in principle, the weekly costs of the MD and Italian household consumption do not differ significantly [24], other findings suggest that greater adherence to the MD increases the monetary diet costs compared with less adherence. In Italy, the Moli-sani study pointed out the role of economic constraints in determining the low adherence to the MD in a period of economic crisis (2007–2010), with greater detrimental effects in the elderly [44]. On the other hand, the role of education may be explained by the influence of nutritional knowledge and awareness about the role of diet in promoting healthy lifestyles, as higher education is generally linked to healthy food consumption [45]. Similar to our results, a recent work carried out on Dutch adults found that highly educated individuals followed better consumption patterns compared with less educated ones [46].

According to the consumption frequency of single food groups, the majority of the respondents were not compliant with national [32] and international [47] dietary recommendations. Indeed, fruit and vegetable intakes were far below the suggested cut-offs (i.e., ≥400 g or ≥5 portions per day). Considering protein-based foods of animal origin, only approximately 30% of the respondents and about 2% among the subjects falling in the low adherence to the MD group declared eating fish or sea food as suggested both by the MD [31] and by the Italian nutritional guidelines [32] (i.e., ≥2 servings per week). Furthermore, the consumption of red meat exceeded 3 portions per week (the cut-off proposed by the Mediterranean dietary pattern) in a low proportion of subjects (<10%). Nevertheless, a higher number of subjects was not compliant with the stricter Italian guidelines, recommending one serving per week of fresh red meat and the occasional consumption of processed or cured meat. The wider inclusion of instances related to environmental sustainability in the national recommendations, being more plant-oriented, explains the discrepancy between the two cut-offs. Our study suggests that among the food components, the lowest level of compliance to the MD-based cut-offs can be observed for olive oil followed by legumes and fish not only in the whole sample of respondents, but also in the highest MD category. This data are in line with previous works, which reported a decrease in olive oil consumption over time [41], probably due to its limited affordability [48].

This study suggests that some changes are needed in the dietary behaviors of Italian adults in order to meet nutritional and environmental guidelines, as is also expressed by the adherence to the MD. From this perspective, public interventions might be defined, for instance, to increase consumers’ awareness of the environmental impact of food choices [49], to nudge the consumer away from bad choices [50], or to modify the relative price of healthy or unhealthy choices with fiscal measures [51,52]. However, as has been suggested by several authors, the public decision-making process might be challenging given the uncertainties regarding outcomes due to the non-linear processes, feedback loops, and trade-offs that occur in food systems [53]. For instance, it has been shown that lowering domestic demand at the European level for meat would affect the profitability of meat production in the EU, in particular the European beef meat sector [54]. The complex network of interdependencies among food systems and dietary behavior outcomes asks for a multidisciplinary approach, requiring concerted efforts between disciplines.

To the best of our knowledge, this is the first study evaluating associations between MD adherence, subjects’ awareness about MD sustainability, and self-perception about their own dietary behavior. Other strengths of this work should be highlighted. First of all, the study population is representative of the Italian adult population in terms of gender, age, and geographical distribution. In addition, important information that may impact food habits was collected (e.g., respondents’ BMIs, nationalities, education levels, incomes, occupations, household sizes, degrees of responsibility for food purchasing and meal preparation, habitual frequency of eating out, and any participation in solidarity purchasing groups or environmental associations). Nevertheless, other potential determinants of dietary habits (e.g., physical activity level and smoking habits) were not investigated. Another limitation of this study is linked to the use of a self-administered online questionnaire, which is a very useful and economical tool but, at the same time, may lead to recall bias and misclassification. In addition, some questions (e.g., the self-perception of the MD’s sustainability and the adoption of a sustainable diet) have not specifically been tested for validity, and this could represent a limitation for the soundness of the results in the present study. On the other hand, the use of a validated questionnaire specifically designed to collect information on adherence to the MD (main outcome) has been used. Furthermore, the distribution of the sample according to certain categorical variables in some case led to a very low number of subjects (e.g., respondents classified as having a little or lack of responsibility in purchasing or preparing food), limiting the analysis reliability.

## 5. Conclusions

The present research appraised MD adherence in a representative sample of adults residing in Italy, investigated the self-perception of adopting a sustainable diet, and expressed subjects’ levels of agreement for considering the MD as a sustainable dietary model. Associations with anthropometric and sociodemographic variables revealed a series of contributing factors to MD adherence, primarily BMI, education attainment, and income level. Overall, the sample reported medium adherence to the MD, which is rooted in Italian culture and a widely recognized model of dietary sustainability. The results suggest a gradual shift away from this dietary pattern and support the need to address efforts for driving dietary transition and developing intervention strategies tailored to a target population of adults. There is a need to spark renewed interest in the general population in rediscovering a traditional dietary model that can boast nutritional, environmental, and social sustainability dimensions. Ensuring economic accessibility to food supplies should be the first public health prevention strategy for improving diet quality and increasing adherence to the MD. Furthermore, public campaigns should stress the link between diet and its environmental impact to foster nutritionally adequate and eco-friendly dietary behaviors.

## Figures and Tables

**Table 1:** Anthropometric variables, demographic and socioeconomic characteristics, health conditions, food-related habits, adherence to the MD, consideration of the MD as a sustainable dietary model, and self-perceived adoption of a sustainable diet in the last 3 months in the total sample, split by gender.

	All (*n* = 838)	Female (*n* = 434)	Male (*n* = 404)	*p* Value
Age range (years)				0.131 ^a^
18–24	89 (10.6)	53 (12.2)	36 (8.9)	
25–34	157 (18.7)	79 (18.2)	78 (19.3)	
35–44	198 (23.6)	111 (25.6)	87 (21.5)	
45–54	209 (24.9)	95 (21.9)	114 (28.2)	
55–65	185 (22.1)	96 (22.1)	89 (22.0)	
BMI (kg/m^2^)				<0.001 ^a^
<18.5 (underweight)	36 (4.3)	33 (7.6)	3 (0.7)	
18.5–24.9 (normal weight)	497 (59.3)	284 (65.4)	213 (52.7)	
25.0–29.9 (overweight)	230 (27.4)	82 (18.9)	148 (36.6)	
≥30.0–34.9 (obesity)	75 (8.9)	26 (6.0)	31 (7.7)	
Health conditions				<0.001 ^a^
Anemia, hypertension, or dyslipidemia	83 (9.9)	36 (8.3)	47 (11.6)	
Food intolerance or allergies	138 (16.5)	70 (16.1)	68 (16.8)	
Menopause, pregnancy, or breastfeeding	70 (8.4)	70 (16.1)	-	
None of the above	547 (65.3)	258 (59.4)	289 (71.5)	
Geographical area of residence				0.683 ^a^
Northwest	220 (26.3)	116 (26.7)	104 (25.7)	
Northeast	168 (20.0)	86 (19.8)	82 (20.3)	
Central	167 (19.9)	85 (19.6)	82 (20.3)	
South	192 (22.9)	94 (21.7)	98 (24.3)	
Islands	91 (10.9)	53 (12.2)	38 (9.4)	
Size of residence (number of inhabitants)				0.300 ^a^
<5000	148 (17.7)	80 (18.4)	68 (16.8)	
5000–49,999	348 (41.5)	188 (43.3)	160 (39.6)	
50,000–500,000	206 (24.6)	95 (21.9)	111 (27.5)	
>500,000	136 (16.2)	71 (16.4)	65 (16.1)	
Education level				0.151 ^a^
Primary or lower secondary	62 (7.4)	26 (6.0)	36 (8.9)	
Secondary	448 (53.5)	243 (56.0)	205 (50.7)	
Tertiary *	328 (39.1)	165 (38.0)	163 (40.3)	
Occupation				<0.001 ^a^
Full-time employee	441 (52.6)	167 (38.5)	274 (67.8)	
Part-time employee	133 (15.9)	93 (21.4)	40 (9.9)	
Unemployed	158 (18.9)	113 (26.0)	45 (11.1)	
Retired	35 (4.2)	16 (3.7)	19 (4.7)	
Student	71 (8.5)	45 (10.4)	26 (6.4)	
Monthly household net income				0.005 ^a^
≤EUR 1499	193 (23.0)	115 (26.5)	78 (19.3)	
EUR 1500–2499	267 (31.9)	132 (30.4)	135 (33.4)	
≥EUR 2500	294 (35.1)	131 (30.2)	163 (40.3)	
Do not wish to tell or do not know	84 (10.0)	56 (12.9)	28 (6.9)	
N. household members				0.084 ^a^
1	76 (9.1)	33 (7.6)	43 (10.6)	
2	182 (21.7)	106 (24.4)	76 (18.8)	
3	267 (31.9)	129 (29.7)	138 (34.2)	
>3	313 (37.4)	166 (38.2)	147 (36.4)	
N. household members < 18 years				0.263 ^a^
None	526 (62.8)	284 (65.4)	242 (59.9)	
1	172 (20.5)	80 (18.4)	92 (22.8)	
2	118 (14.1)	57 (13.1)	61 (15.1)	
≥3	22 (2.6)	13 (3.0)	9 (2.2)	
Responsibility of food purchases				<0.001 ^a^
Mainly responsible	601 (71.7)	347 (80.0)	254 (62.9)	
Co-responsible	222 (26.5)	84 (19.4)	138 (34.2)	
Little or not at all responsible	15 (1.8)	3 (0.7)	12 (3.0)	
Responsibility in meal preparation				<0.001 ^a^
Mainly responsible	529 (63.1)	346 (79.7)	183 (45.3)	
Co-responsible	262 (31.3)	78 (18.0)	184 (45.5)	
Little or not at all responsible	47 (5.6)	10 (2.3)	37 (9.2)	
Frequency of eating out				<0.001 ^a^
Never or seldom	129 (15.4)	83 (19.1)	46 (11.4)	
<1 time/week	167 (19.9)	101 (23.3)	66 (16.3)	
1 time/week	195 (23.3)	101 (23.3)	94 (23.3)	
2–4 times/week	249 (29.7)	117 (27.0)	132 (23.7)	
≥5 times/week	98 (11.7)	32 (7.4)	66 (16.3)	
Taking part in SPGs or EAs				0.634 ^a^
Yes	93 (11.1)	46 (10.6)	47 (11.6)	
No	745 (88.9)	388 (89.4)	357 (88.4)	
MD score (on a 0–9 scale)	4.0 (3.0–5.0)	4.0 (3.0–6.0)	3.0 (2.0–5.0)	<0.001 ^b^
Adherence to the Mediterranean Diet				<0.001 ^a^
Low	164 (19.6)	52 (12.0)	112 (27.7)	
Medium	498 (59.4)	261 (60.1)	237 (58.7)	
High	176 (21.0)	121 (27.9)	55 (13.6)	
MD considered a sustainable dietary model				0.375 ^a^
No	10 (1.2)	3 (0.7)	7 (1.7)	
Maybe	410 (48.9)	212 (48.8)	198 (49.0)	
Yes	418 (49.9)	219 (50.5)	199 (49.3)	
Self-perceived adoption of a sustainable diet within the last 3 months				0.317 ^a^
No	175 (20.9)	85 (19.6)	90 (22.3)	
Not much	526 (62.8)	283 (65.2)	243 (60.1)	
Yes	137 (16.3)	66 (15.2)	71 (17.6)	

Note: * including short cycle tertiary education. Data are expressed as a number (%) or as the median (IR). ^a^ Chi-square test. ^b^ Non-parametric Mann–Whitney test for independent sample. EAs: environmental associations; SPGs: solidarity purchasing groups.

**Table 2:** Compliance with food consumption recommendations used to compute the MD score (MD) and following the Italian guidelines (IT) for healthy eating for a standard dietary energy intake of 2000 kcal/day, according to the level of adherence to the MD.

	Ref. Intake	Adherence to the MD			
		Low (*n* = 164)	Medium (*n* = 498)	High (*n* = 176)	*p* Value
Wholegrains	MD (≥1/d)	87 (53.0)	417 (83.7)	158 (89.8)	<0.001
	IT (n.a.)	n.a.	n.a.	n.a.	
Vegetables	MD (≥2/d)	3 (1.8)	121 (24.3)	137 (77.8)	<0.001
	IT (≥2/d)	3 (1.8)	121 (24.3)	137 (77.8)	<0.001
Fruit	MD (≥2/d)	8 (4.9)	188 (37.8)	150 (85.2)	<0.001
	IT (≥3/d)	–	33 (6.6)	41 (23.3)	<0.001
Milk and yogurt	MD (n.a.)	n.a.	n.a.	n.a.	
	IT (≥3/d)	1 (0.6)	8 (1.6)	8 (4.5)	0.021
Olive oil	MD (≥3/d)	1 (0.6)	46 (9.2)	64 (36.4)	<0.001
	IT (3–4/d)	1 (0.6)	41 (8.2)	54 (30.7)	<0.001
Butter, margarine, or cooking cream	MD (n.a.)	n.a.	n.a.	n.a.	
	IT (<3/d)	163 (99.4)	488 (98.0)	170 (96.6)	0.187
Wine	MD (1–2/d, M; >0 < 1/d, F)	44 (26.8)	291 (58.4)	134 (76.1)	<0.001
	IT (never or hardly never)	81 (49.4)	236 (47.4)	88 (50.0)	0.800
Red meat or meat products	MD (≤3/w)	125 (76.2)	461 (92.6)	173 (98.3)	<0.001
	IT (≤1/w)	53 (32.3)	183 (36.7)	94 (53.4)	<0.001
White meat	MD (n.a.)	n.a.	n.a.	n.a.	
	IT (1–3/w)	84 (51.2)	296 (59.4)	97 (55.1)	0.158
Carbonated or SSB	MD	n.a.	n.a.	n.a.	
	IT (<1/w)	108 (65.9)	346 (69.5)	142 (80.7)	0.005
Sweets	MD	n.a.	n.a.	n.a.	
	IT (<1/w)	103 (62.8)	295 (59.2)	112 (63.6)	0.501
Fish or seafood	MD (≥2/w)	4 (2.4)	155 (31.1)	115 (65.3)	<0.001
	IT (2–3/w)	3 (1.8)	146 (29.3)	104 (59.1)	<0.001
Nuts	MD (≥2/w)	3 (1.8)	123 (24.7)	117 (66.5)	<0.001
	IT (2–3/w)	2 (1.2)	89 (17.9)	70 (39.8)	<0.001
Pulses	MD (≥2/w)	7 (4.3)	120 (24.1)	111 (63.1)	<0.001
	IT (2–3/w)	7 (4.3)	112 (22.5)	96 (54.5)	<0.001

Note: data are expressed as a number (%). The recommended food consumption frequency on a daily (d) or weekly (w) basis of the reference serving(s) are shown in the second column. The reported national reference intake for fruit and milk or yogurt (≥3 instead of 3 servings/day), olive oil (3–4 instead of 3 servings/day) butter, margarine, or cooking cream (<3 instead of ≤3 servings/day), white meat (1–3 instead of 2 servings/day), pulses (2–3 instead of 3 servings/week), and nuts (2–3 instead of 2 servings/week) have been slightly adapted to equal the categorization of the food consumption frequency provided by the MD questionnaire. Chi-square test. Low = first tertile; Medium = second tertile; High = third tertile; SSB: sugar-sweetened beverages.

**Table 3:** Logistic regression analysis for being in the third tertile of distribution of the adherence score to the MD (6-9 points) by considering all the assessed variables alone (univariate analysis) or together (multivariate analysis).

	Univariate Analysis		Multivariate Analysis	
Variables	OR (95% CI)	*p* Value	OR (95% CI)	*p* Value
Gender				
Females	-1-		-1-	
Males	0.408 (0.286–0.580)	<0.001	0.366 (0.220–0.609)	<0.001
Age (years)				
18–24	-1-		-1-	
25–34	0.948 (0.479–1.873)	0.877	0.459 (0.164–1.287)	0.139
35–44	1.342 (0.711–2.532)	0.364	0.704 (0.251–1.979)	0.506
45–54	1.182 (0.625–2.234)	0.607	0.764 (0.275–2.123)	0.606
55–65	1.467 (0.776–2.772)	0.239	0.667 (0.222–2.002)	0.470
BMI (kg/m^2^)				
<18.5	-1-		-1-	
18.5–24.9	0.623 (0.302–1.283)	0.199	0.693 (0.265–1.811)	0.454
25.0–29.9	0.383 (0.176–0.834)	0.016	0.632 (0.225–1.774)	0.383
≥30.0	0.273 (0.102–0.728)	0.010	0.647 (0.187–2.235)	0.491
Health conditions				
Anemia, hypertension, or dyslipidemia	1.180 (0.663–2.100)	0.573	1.062 (0.503–2.242)	0.875
Food intolerance or allergies	1.498 (0.960–2.336)	0.075	1.558 (0.909–2.669)	0.106
Menopause, pregnancy, or breastfeeding	2.877 (1.696–4.880)	<0.001	1.924 (0.916–4.042)	0.084
None of the above	-1-		-1-	
Geographical area of residence				
Northwest	-1-		-1-	
Northeast	0.986 (0.608–1.599)	0.953	1.008 (0.537–1.893)	0.980
Central	0.925 (0.567–1.510)	0.756	0.840 (0.457–1.544)	0.574
South	0.918 (0.573–1.471)	0.723	1.109 (0.605–2.033)	0.738
Islands	0.689 (0.364–1.304)	0.252	0.635 (0.280–1.438)	0.276
Size of residence (number of inhabitants)				
<5000	-1-		-1-	
5000–49,999	1.160 (0.705–1.911)	0.559	0.880 (0.471–1.643)	0.687
50,000–500,000	1.166 (0.677–2.009)	0.580	0.876 (0.439–1.749)	0.707
>500,000	1.955 (1.115–3.428)	0.019	1.622 (0.791–3.324)	0.187
Educational level				
Primary or lower secondary	-1-		-1-	
Secondary	2.946 (1.148–7.560)	0.025	3.098 (1.020–9.410)	0.046
Tertiary * or higher	3.617 (1.401–9.339)	0.008	3.072 (0.973–9.700)	0.056
Occupation				
Full-time employee	-1-		-1-	
Part-time employee	1.186 (0.753–1.867)	0.461	1.372 (0.737–2.551)	0.318
Unemployed	0.741 (0.462–1.187)	0.213	1.041 (0.514–2.107)	0.912
Retired	1.437 (0.667–3.097)	0.354	1.961 (0.706–5.451)	0.196
Student	0.589 (0.291–1.193)	0.142	0.599 (0.181–1.982)	0.401
Monthly household net income				
≤EUR 1499	-1-		-1-	
EUR 1500–2499	1.705 (1.026–2.832)	0.039	1.950 (1.051–3.620)	0.034
≥EUR 2500	2.160 (1.324–3.527)	0.002	2.419 (1.225–4.777)	0.011
Number of household members				
1	-1-		-1-	
2	1.195 (0.594–2.405)	0.618	0.890 (0.364–2.178)	0.798
3	1.623 (0.841–3.135)	0.149	1.880 (0.748–4.725)	0.180
>3	1.149 (0.594–2.224)	0.679	0.722 (0.261–1.995)	0.529
Number of household members <18 years				
None	-1-		-1-	
1	1.179 (0.774–1.794)	0.443	0.576 (0.312–1.065)	0.079
2	1.417 (0.888–2.261)	0.144	1.789 (0.793–4.033)	0.161
≥3	1.559 (0.595–4.083)	0.366	1.970 (0.549–7.060)	0.298
Responsibility of food purchases #				
Main responsible	-1-		-1-	
Co-, little, or not at all responsible	0.495 (0.326–0.752)	0.001	0.905 (0.446–1.836)	0.781
Responsibility in food preparation				
Main responsible	-1-		-1-	
Co-responsible	0.516 (0.348–0.763)	0.001	0.875 (0.453–1.690)	0.690
Little or not at all responsible	0.201 (0.061–0.658)	0.008	0.748 (0.170–3.287)	0.700
Frequency of eating out				
Never or seldom	-1-		-1-	
<1 time/week	1.648 (0.897–3.029)	0.108	1.730 (0.815–3.674)	0.154
1 time/week	1.737 (0.963–3.133)	0.067	1.906 (0.903–4.022)	0.091
2–4 times/week	1.641 (0.927–2.907)	0.089	1.405 (0.649–3.039)	0.388
≥5 times/week	1.484 (0.743–2.965)	0.263	1.317 (0.534–3.252)	0.550
Taking part in SPGs or EAs				
Yes	-1-		-1-	
No	0.650 (0.399–1.057)	0.083	1.038 (0.557–1.936)	0.906
MD considered a sustainable dietary model #				
No/maybe	-1-		-1-	
Yes	2.617 (1.840–3.723)	<0.001	2.293 (1.487–3.534)	<0.001
Self-perceived adoption of a sustainable diet				
No	-1-		-1-	
Not much	3.388 (1.815–6.323)	<0.001	2.162 (1.089–4.293)	0.028
Yes	10.275 (5.222–20.216)	<0.001	7.667 (3.517–16.711)	<0.001

Note: * including short cycle tertiary education. # The categories “No” and “Maybe”, as well as the categories “Co-responsible” and “Little or not at all responsible” have been collapsed into one due to the lack of subjects in single categories or tertiles. EA: environmental association; SPGs: solidarity purchasing groups.

## Data Availability

The data presented in this study are available on request from the corresponding author.

## Supplementary Materials

The following are available online at https://www.mdpi.com/article/10.3390/nu13093282/s1, Table S1: Percentage distribution of respondents according to their consumption frequency of single food groups/items.
