# Supplementary material for: Adherence to the Mediterranean Diet in Association with Self-Perception of Diet Sustainability, Anthropometric and Sociodemographic Factors: A Cross-Sectional Study in Italian Adults
Source: Nutrients. 2021 Sep 20;13(9):3282. doi: 10.3390/nu13093282 (PMC8468784; doi:10.3390/nu13093282)
Supplement: Supplementary file 1 [file nutrients-13-03282-s001.zip › nutrients-1340361-supplementary.pdf]

Supplementary Table S1. Percentage distribution of respondents according to their consumption frequency of single food groups/items.

	Percentage of respondents (n= 838)				
Food groups	Never or seldom	<1/day	1/day	2/day	≥3/day
Wholegrain pasta or rice	19.2	42.2	33.4	4.1	1.1
Vegetables, all types	8.7	26.8	33.3	28.0	3.1
Fruit, all types fresh and fresh juices	7.2	25.4	26.1	32.5	8.8
Milk and yoghurt	19.9	30.0	36.9	11.2	2.0
	Never or seldom	<1/day	1-2/day	3-4/day	≥5/day
Wholegrain bread and substitutes	22.0	33.1	39.9	4.4	0.7
Olive oil to cook and to dress	4.2	22.8	59.8	11.5	1.8
Butter, margarine or cooking cream	58.4	32.5	7.2	1.4	0.6
Wine	48.3	30.8	17.3	2.9	0.7
	Never or seldom	<1/week	1-3/week	4-6/week	≥7/week
Red meat, meat products	11.7	27.7	51.2	8.1	1.3
White meat	9.1	28.3	56.9	4.9	0.8
Carbonated and/or SSB	43.3	27.8	21.5	4.5	2.9
Sweets	23.5	37.4	24.0	10.1	5.0
	Never or seldom	<1/week	1/week	2-3/week	≥4/week
Fish or seafood	9.7	28.9	28.8	30.2	2.5
Nuts	30.2	26.1	14.7	19.2	9.8
Pulses	12.5	30.4	28.6	25.7	2.7

Note: The categories of consumption frequencies are expressed as number of servings per day or week. SSB: sugar-sweetened beverages.
